# TSH suppression attenuates the early efficacy of zoledronic acid in osteoporosis

**DOI:** 10.1007/s11657-026-01672-2

**Published:** 2026-02-24

**Authors:** Yujuan Liu, Yilei Zhang, Meiye Li, Ying Qian, Zongjing Zhang, Zhaoshun Jiang, Wei Qu

**Affiliations:** 1 Shandong First Medical University, Jinan, People’s Republic of China; 2 The 960th Hospital of the PLA Joint Logistics Support Force, Jinan, People’s Republic of China

**Keywords:** TSH suppression therapy, Osteoporosis, Bone mineral density, Differentiated thyroid cancer, Zoledronic acid

## Abstract

***Summary*:**

TSH suppression was associated with an attenuated early skeletal response to zoledronic acid in postoperative DTC patients with osteoporosis.

**Objective:**

This prospective study aimed to evaluate the impact of TSH suppression therapy on the early skeletal efficacy of zoledronic acid in patients with differentiated thyroid cancer (DTC), and to explore whether this response differs across subgroups.

**Methods:**

Patients were divided into two groups: the osteoporosis with TSH suppression group (TSH + OP, n = 42) and the osteoporosis without TSH suppression group (TSH − OP, n = 67). Both groups received a single infusion of zoledronic acid (5 mg) along with daily calcium and calcitriol supplementation. Primary analyses compared changes in bone mineral density (BMD) and bone turnover markers(BTMs) at 6 and 9 months post-treatment. After propensity score matching (PSM), 36 patients from each group were included in the primary analyses. Exploratory subgroup analyses were conducted according to age and sex.

**Results:**

After PSM, the TSH + OP group showed significantly less BMD improvement compared to the TSH − OP group. At 9 months, the intergroup differences in BMD change were -7.08 mg/cm^2^ (lumbar, P = 0.02), -12.03 mg/cm^2^ (left hip, P < 0.001) and -6.12 mg/cm^2^ (femoral neck, P < 0.001). BTMs (PINP and β-CTX) remained higher in the TSH + OP group at all time points (all P < 0.01). Subgroup analyses suggested that these differences were primarily observed in postmenopausal patients.

**Conclusion:**

TSH suppression therapy was associated with an attenuated early skeletal response to zoledronic acid in postoperative DTC patients with osteoporosis, with this attenuation being more pronounced at the hip and femoral neck. Exploratory subgroup analyses suggested that this attenuation was primarily observed in postmenopausal women. In patients undergoing TSH suppression, serum PINP and β-CTX are useful monitoring biomarkers.

## Introduction

In recent years, the global incidence of thyroid cancer has been steadily increasing. According to the 2022 Global Cancer Statistics published by the International Agency for Research on Cancer, thyroid cancer ranks seventh among all malignant tumors. Differentiated thyroid cancer (DTC) is the most prevalent type, accounting for approximately 90% of cases. Advanced imaging techniques enable earlier detection of thyroid cancer, yet may lead to overtreatment in low-risk patients through total thyroidectomy, radioactive iodine ablation, and lifelong levothyroxine therapy. While essential for preventing recurrence, aggressive TSH suppression can cause subclinical hyperthyroidism with adverse effects including atrial fibrillation and osteoporosis, necessitating careful risk–benefit consideration [1–3]. Numerous studies have established a strong correlation between TSH levels and bone mineral density (BMD). For example, Ding et al. further noted that lower TSH concentrations in elderly women correlate with decreased femoral neck BMD, indicating that individuals with low TSH levels may experience a higher incidence of osteoporosis and diminished bone mass [4]. Kim et al. found that dual-energy X-ray absorptiometry (DXA) has lower sensitivity than quantitative CT (cQCT) in diagnosing bone mass reduction and osteoporosis following TSH suppression therapy. This difference leads to a reduced detection rate of bone loss [5]. Einspieler et al. observed via CT that prolonged TSH suppression correlated with a significant reduction in BMD; however, this finding was not consistently confirmed by DXA measurements [6, 7]. Moreover, numerous studies have demonstrated that both the intensity and duration of TSH suppression are significantly correlated with a reduction in BMD in postmenopausal women. Elevated thyroid hormone levels can shorten the bone remodeling cycle, increase bone turnover, and consequently result in decreased bone density [8, 9]. Panico et al. examined the efficacy of alendronate sodium in postmenopausal women, comparing those who received levothyroxine treatment with those who did not. They found that as the duration of TSH suppression increased, the effectiveness of alendronate sodium in enhancing BMD diminished progressively [10]. Multiple lines of evidence consistently demonstrate that both exogenous and endogenous subclinical hyperthyroidism are significantly linked to reduced bone density and an elevated risk of fractures, particularly in the femoral neck region [11–14].

However, the relationship between TSH suppression therapy and BMD remains controversial, as different detection methods and research contexts yield inconsistent conclusions. Furthermore, most relevant studies are limited to observations in postmenopausal women, and therefore a clinical gap remains due to a lack of research that includes younger and male patients. Whether TSH suppression therapy modifies the skeletal response to anti-resorptive treatment remains poorly understood. In particular, it is unclear whether patients with osteoporosis receiving TSH suppression exhibit a different early skeletal response to zoledronic acid compared with those with osteoporosis not exposed to TSH suppression, and whether such effects vary across subgroups.

Accordingly, this prospective study aimed to evaluate the impact of TSH suppression therapy on the early skeletal efficacy of zoledronic acid in patients with differentiated thyroid cancer and osteoporosis.

### Study design

This study employed a prospective interventional cohort design. Patients with DTC who underwent total thyroidectomy at the 960th Hospital of the Joint Logistic Support Force of the People's Liberation Army of China between 2015 and 2025 were consecutively recruited. All patients were actively followed up, and those who completed bone density tests and provided informed consent were included in the study cohort.

### Study participants

Inclusion criteria: (1) Age ≥ 20 years and completion of baseline BMD assessment; (2) osteopenia or osteoporosis defined according to age-appropriate criteria. In participants aged ≥ 50 years, osteoporosis was defined as a T-score ≤ − 2.5 at the lumbar spine, femoral neck, or total hip, and osteopenia as a T-score between − 1.0 and − 2.5, in accordance with World Health Organization(WHO) criteria. In participants aged < 50 years, because formal WHO diagnostic thresholds for osteoporosis and osteopenia are not established, operational definitions were applied for study stratification. Marked bone mass loss was defined as a Z-score ≤ − 2.0; these individuals were assigned to the osteoporosis group. Participants with Z-scores between − 1.0 and − 2.0 were classified as having osteopenia and were assigned to the osteopenia group; (3) ability to complete follow-up and provide informed consent. Additional criteria for the TSH + OP and TSH + OPE groups: confirmed DTC treated with total thyroidectomy and receiving TSH suppression therapy for ≥ 6 months. Exclusion criteria: type 1 diabetes, parathyroid dysfunction, endocrine/metabolic disorders (e.g., kidney disease, hypopituitarism, Cushing's syndrome), autoimmune diseases, neoplastic conditions, ongoing anti-osteoporosis treatment, use of bone metabolism-affecting medications, pregnancy or planning pregnancy, and unwillingness to cooperate.

**Fig. 1:**
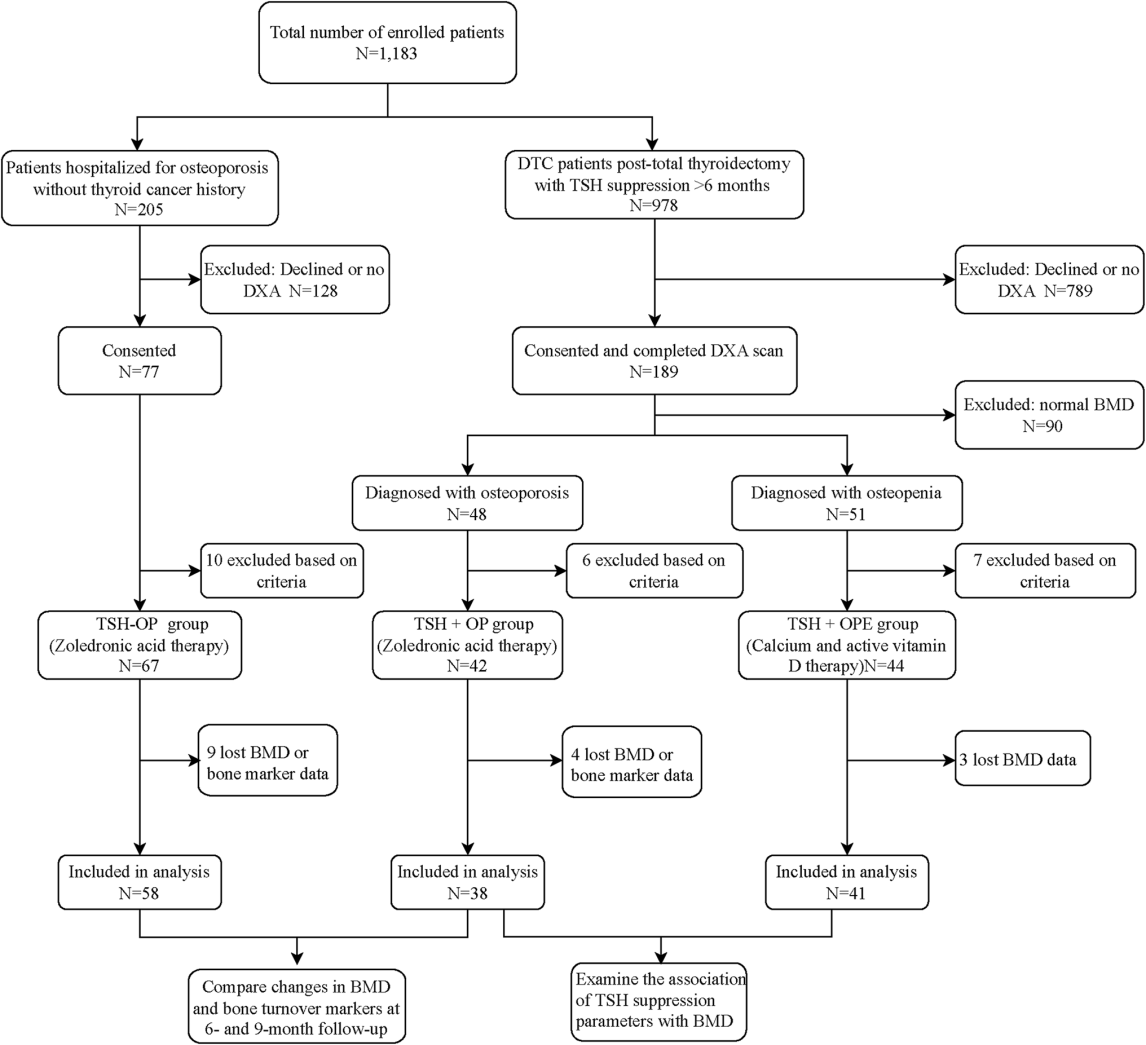
Flowchart of patient screening, enrollment, and grouping

A total of 1,183 individuals from two distinct source populations were screened for eligibility. These included 978 consecutive patients with differentiated thyroid cancer who underwent total thyroidectomy at our hospital between 2015 and 2025, and 205 patients with osteoporosis who were hospitalized at our institution between 2024 and 2025 and had no history of thyroid cancer. Among these 1,183 individuals, 266 patients consented to participate in the study and completed at least one baseline BMD assessment. After excluding individuals with normal BMD (n = 90), those with combined parathyroid dysfunction (*n* = 2), kidney disease (*n* = 9), current glucocorticoid use (*n* = 7), or concurrent malignant tumors (*n* = 5), the remaining patients were categorized based on their receipt of TSH suppression therapy and BMD status. This resulted in three groups: patients with osteoporosis receiving TSH suppression therapy (TSH + OP group, *n* = 42), patients with osteoporosis not receiving TSH suppression therapy (TSH − OP group, *n* = 67), and patients with osteopenia receiving TSH suppression therapy (TSH + OPE group, *n* = 44). The patient screening and grouping processes are illustrated in Fig. 1. No participants with a history of fragility fracture were included. All patients classified as osteoporotic met densitometric criteria. All patients with DTC underwent total thyroidectomy followed by radioactive iodine ablation, and no evidence of distant metastases was observed. All female patients were in a natural postmenopausal state, having experienced regular menstrual cycles prior to menopause and lacking any history of prior oophorectomy. This study complied with the principles outlined in the Declaration of Helsinki and was approved by the Ethics Review Committee of the 960th Hospital of the Joint Logistic Support Force of the People's Liberation Army of China (Approval No.2024–126).

### Study intervention

The TSH + OP and TSH − OP groups received a single intravenous infusion of 5 mg zoledronic acid, along with daily oral administration of 600 mg calcium carbonate and 0.5 μg calcitriol. The initiation of zoledronic acid therapy was based on the Chinese Guidelines for the Diagnosis and Treatment of Primary Osteoporosis and was guided by BMD–based risk stratification. Specifically, zoledronic acid was administered to participants aged ≥ 50 years who met the WHO diagnostic criteria for osteoporosis (T-score ≤ − 2.5 at the lumbar spine, femoral neck, or total hip), as well as to participants aged < 50 years who exhibited markedly reduced bone mass (Z-score ≤ − 2.0) under long-term TSH suppression therapy [15]. In contrast, bisphosphonate drugs were administered only once, whereas calcium and active vitamin D supplementation continued throughout the study follow-up. All adverse events were documented; however, all patients were included in the final efficacy analysis, regardless of the occurrence of adverse reactions.

### Data collection

Data were collected through standardized questionnaires and laboratory tests, capturing demographic/clinical characteristics (age, sex, BMI, menopausal status, lifestyle factors, comorbidities, TSH suppression duration) and laboratory parameters (thyroid function, lumbar and left hip BMD, PINP, β-CTX, vitamin D, and other biochemical markers). BMD and BTMs were measured at baseline, 6, and 9 months in TSH + OP and TSH − OP groups, while only baseline BMD was assessed in the TSH + OPE group.

### Bone mineral density

BMD was measured at the lumbar spine and left hip using dual-energy X-ray absorptiometry (Lunar-Prodigy, GE, USA), and all scans were performed by a consistent team of technicians. Daily calibration was performed using a manufacturer-supplied phantom to ensure data quality. Osteoporosis and osteopenia were diagnosed according to WHO criteria, and T-scores were calculated using the device’s built-in Asian reference database. A precision assessment conducted prior to the study revealed in vivo coefficients of variation of 1.2% for lumbar spine BMD, 1.8% for left hip BMD, and 2.0% for femoral neck BMD at our center, corresponding to least significant changes of 3.3%, 5.0% and 5.5%, respectively.

### Statistical analysis

All statistical analyses were performed using R software (Version 4.5.1). Continuous variables were reported as mean ± standard deviation. Independent sample t-tests or Mann–Whitney U tests were employed for group comparisons, depending on the data distribution. Categorical variables were presented as frequencies and percentages, with chi-square tests or Fisher's exact tests used for intergroup comparisons.

To control for baseline confounding factors between the TSH + OP and TSH − OP groups, we constructed a balanced cohort using 1:1 propensity score matching (PSM) without replacement. The propensity score was estimated from a model that included age, sex, BMI, menopausal status, smoking, and drinking status. Matching was performed using a caliper width of 0.2 standard deviations. PSM substantially improved the balance between groups. After matching, the standardized mean differences (SMDs) for age, sex, BMI, menopausal status, and smoking history were all reduced to below 10%, indicating excellent balance for these key covariates. The SMD for alcohol use was reduced from 19.8% to 10.3%. Overall, the model’s pseudo R^2^ decreased dramatically from 0.067 to 0.003, and the mean bias across all covariates was reduced from 23.3% to 2.9%, collectively confirming the effectiveness of the matching procedure in minimizing baseline confounding. After matching, 36 patients from each group were included in the primary analysis, which focused on comparing early changes in BMD and bone turnover markers between the TSH + OP and TSH − OP groups following zoledronic acid treatment.

For the primary analysis, a linear mixed-effects model was used to assess the impact of treatment on BMD and bone metabolism indicators. This model incorporated treatment groups, time, and their interaction terms as fixed effects while adjusting for age, sex, and body mass index as covariates. Additionally, individual random intercepts for the subjects were included to account for the correlation inherent in the repeated measurement data.

Exploratory subgroup analyses were conducted in the matched cohort by stratifying patients according to sex (male vs female) and age (< 50 vs ≥ 50 years). Within each stratum, linear mixed-effects models similar to the primary analysis were fitted to evaluate treatment-by-time interactions, with adjustment for the remaining covariates as appropriate.

Finally, in exploratory analyses, using multiple linear regression with appropriate corrections for relevant covariates, we examined the relationship between TSH suppression parameters and BMD in the combined TSH + OP and TSH + OPE groups. All tests were two-tailed, and a *P*-value of less than 0.05 was deemed statistically significant.

## Results

The primary analysis focused on early changes in BMD and BTMs following zoledronic acid treatment in the matched TSH + OP and TSH − OP groups. This study analyzed 153 patients (62 men, 91 women; mean age 56.55 ± 15.87 years) distributed into TSH + OP (*n* = 42), TSH − OP (*n* = 67), and TSH + OPE (*n* = 44) groups. Data were missing for 16 patients. Ultimately, 38 patients in the TSH + OP group, 58 patients in the TSH − OP group, and 41 patients in the TSH + OPE group completed all follow-ups and provided complete datasets. PSM (1:1) created balanced TSH + OP group and TSH − OP group (*n* = 36 each) with comparable baseline characteristics except for serum TSH and FT4 levels (Table 1). The baseline data of patients in the TSH + OPE group are presented in Table 2. After PSM, the T/Z scores and BMD values at baseline, 6 months, and 9 months are presented in Table 3.

**Table 1:** Baseline characteristics of patients with OP, with and without TSH suppression, before and after propensity score matching

Characteristics	Before matching			After matching		
	TSH + OP group (*n* = 38)	TSH − OP group (*n* = 58)	*P* value	TSH + OP group (*n* = 36)	TSH − OP group (*n* = 36)	*P* value
**Matched variables**						
Age, mean (SD), year	52.74 (15.54)	60.66 (16.27)	0.02	54.03 (14.88)	54.78 (16.39)	0.95
Male	39.18 (12.20)	46.90 (19.02)		40.47 (12.33)	41.60 (17.12)	
Female	63.71 (6.68)	67.89 (8.11)		63.71 (6.68)	64.19 (6.45)	
Sex, *n* (%)			0.31			1.00
Male	17 (44.74)	20 (34.48)		15 (41.67)	15 (41.67)	
Female	21 (55.26)	38 (65.52)		21 (58.33)	21 (58.33)	
BMI, mean, kg/m^2^	24.85 (3.18)	23.94 (3.95)	0.24	24.76 (3.21)	24.68 (3.91)	0.93
Menopause, *n* (%)			1.00			1.00
Yes	21 (100.00)	38 (100.00)		21 (100.00)	21 (100.00)	
No	0 (0.00)	0 (0.00)		0 (0.00)	0 (0.00)	
Smoke, *n* (%)	7 (18.42)	10 (17.24)	0.88	7 (19.44)	7 (19.44)	1.00
Drink, *n* (%)	4 (10.53)	3 (5.17)	0.43	4 (11.11)	3 (8.33)	1.00
**Other variables, mean (SD)**						
LS BMD, mg/cm^2^	949.35 (207.43)	861.51 (175.41)	0.03	933.60 (195.77)	878.50 (187.78)	0.23
LH BMD, mg/cm^2^	747.32 (94.59)	701.38 (89.07)	0.02	743.22 (94.37)	715.96 (87.08)	0.21
FN BMD, mg/cm^2^	706.98 (96.95)	669.13 (91.90)	0.04	699.92 (94.37)	663.03 (90.14)	0.34
TSH, IU/mL	0.13 (0.17)	1.74 (0.0.76)	0.00	0.13 (0.17)	1.76 (0.80)	0.00
FT4, pmol/L	18.88 (3.05)	17.25 (1.97)	0.01	18.88 (3.14)	17.06 (2.06)	0.01
FT3, pmol/L	4.76 (0.76)	4.71 (0.68)	0.54	4.76 (0.77)	4.61 (0.62)	0.35
Ca, mmol/L	2.29 (0.12)	2.28 (0.09)	0.62	2.28 (0.12)	2.27 (0.09)	0.60
P, mmol/L	1.16 (0.18)	1.19 (0.17)	0.47	1.17 (0.18)	1.19 (0.17)	0.56
AKP, U/L	89.08 (29.99)	91.31 (28.84)	0.72	90.75 (29.93)	87.44 (30.61)	0.65
25 (OH)D, ng/mL	19.69 (7.60)	22.34 (7.18)	0.09	19.67 (7.81)	21.23 (6.84)	0.37
PINP, ng/mL	58.67 (13.20)	62.78 (16.33)	0.25	58.44 (12.90)	61.36 (17.61)	0.43
β-CTX, μg/L	0.55 (0.20)	0.62 (0.21)	0.08	0.55 (0.20)	0.63 (0.23)	0.10
OST, μg/L	21.07 (8.83)	18.53 (7.54)	0.14	21.07 (9.04)	19.11 (8.18)	0.29
PTH, pg/mL	35.82 (10.83)	32.10 (10.58)	0.10	35.45 (10.74)	32.45 (8.61)	0.20
ALT, U/L	19.00 (9.24)	16.91 (8.65)	0.23	18.97 (9.37)	18.00 (9.32)	0.63
AST, U/L	17.63 (4.61)	17.78 (4.72)	0.99	17.64 (4.62)	17.64 (3.99)	0.88
ALB, g/L	42.22 (3.34)	40.99 (3.48)	0.03	42.44 (3.47)	41.11 (3.94)	0.13
Cr, μmol/L	59.24 (12.42)	61.10 (15.71)	0.60	59.31 (12.77)	60.92 (15.41)	0.71
BUN, mmol/L	5.19 (1.04)	5.40 (1.26)	0.39	5.12 (1.01)	5.17 (1.22)	0.96
Comorbidities, *n* (%)						
Hypertension	8 (21.05)	8 (13.79)	0.35	8 (22.22)	4 (11.11)	0.21
Diabetes	9 (23.68)	8 (13.79)	0.21	9 (25.00)	4 (11.11)	0.13
Coronary heart disease	3 (7.89)	1 (1.72)	0.30	3 (8.33)	0 (0.00)	0.24
Hyperlipemia	4 (10.53)	3 (5.17)	0.43	3 (8.33)	1 (2.78)	0.61
TSH suppression time, mean(SD), year	3.26(2.11)			3.35(2.12)		

Data were presented as mean ± SD for continuous variables and number (percentage) for categorical variables.

*BMI* body mass index, *BMD* bone mineral density, *LS BMD* lumbar spine BMD, *LH BMD* left hip BMD, *FN BMD* femoral neck BMD, *TSH* thyroid stimulating hormone, *FT4* free thyroxine, *FT3* free triiodothyronine, *Ca* calcium, *P* phosphorus, *AKP* alkaline phosphatase, *25(OH)D* 25-hydroxyvitamin D, *PINP* N-terminal propeptide of type I procollagen, *β-CTX* β-C-terminal telopeptide of type I collagen, *OST* osteocalcin, *PTH* parathyroid hormone, *ALT* alanine aminotransferase, *AST* aspartate aminotransferase, *ALB* albumin, *Cr* creatinine, *BUN* blood urea nitrogen

**Table 2:** Baseline characteristics of patients in the TSH + OPE group

Characteristics	TSH + OPE group
Age, mean (SD), year	51.27 (14.38)
Age, n (%)	
≥ 50 years	56.96
< 50 years	43.04
Sex, n (%)	
Male	23
Female	18
BMI, mean, kg/m^2^	24.09 (2.79)
Menopause, n (%)	
Yes	41 (100)
No	0 (0.00)
Smoke, n (%)	4 (9.76)
Drink, n (%)	3 (7.32)
**Other variables, mean (SD)**	
LS BMD T/Z score	−0.69 (0.81)
LS BMD, mg/cm^2^	1101.94 (156.43)
LH BMD T/Z score	−1.45 (0.51)
LH BMD, mg/cm^2^	851.21 (73.33)
FN BMD T/Z score	−0.86 (0.59)
FN BMD, mg/cm^2^	826.25 (83.13)
TSH, IU/mL	0.19 (0.31)
FT4, pmol/L	20.42 (3.52)
FT3, pmol/L	4.74 (0.66)
Ca, mmol/L	2.32 (0.12)
P, mmol/L	1.20 (0.19)
AKP, U/L	86.88 (25.81)
25(OH)D, ng/mL	21.12 (5.35)
PINP, ng/mL	49.29 (11.32)
β-CTX, μg/L	0.45 (0.20)
OST, μg/L	18.85 (5.77)
PTH, pg/mL	35.13 (11.96)
Comorbidities, n (%)	
Hypertension	8 (19.51)
Diabetes	11 (26.83)
Coronary heart disease	5 (12.20)
Hyperlipemia	5 (12.20)
TSH suppression time, mean (SD), year	2.89 (1.86)

In the "T/Z score" row, data for participants aged ≥ 50 years represent their T-scores, while data for participants aged < 50 years represent their Z-scores. This row displays the combined mean of both scores

**Table 3:** T/Z scores and absolute BMD values at baseline, 6 and 9 months for the propensity-score matched cohort

	Proportion of aged ≥ 50 years	LS BMD T/Z score	LS BMD, mg/cm^2^	LH BMD T/Z score	LH BMD, mg/cm^2^	FN BMD T/Z score	FN BMD, mg/cm^2^
TSH + OP group							
Baseline	61.11%	−2.03(1.26)	933.60 (195.77)	−2.60(0.53)	743.22 (94.37)	−2.71(0.44)	699.92 (94.37)
6 month	61.11%	−1.89(1.25)	946.61 (201.48)	−2.40(0.47)	758.52 (92.94)	−2.54(0.38)	711.89 (91.82)
9 month	61.11%	−1.80(1.28)	958.52 (208.60)	−2.27(0.53)	766.71 (99.74)	−2.42(0.41)	722.50 (93.97)
TSH − OP group							
Baseline	69.44%	−2.15(0.87)	878.50 (187.78)	−2.51(0.4)	715.96 (87.08)	−2.64(0.23)	663.03 (90.14)
6 month	69.44%	−1.92(0.87)	893.69 (186.57)	−2.25(0.38)	735.66 (90.89)	−2.45(0.25)	692.52 (89.17)
9 month	69.44%	−1.73(0.89)	910.91 (185.76)	−2.06(0.43)	750.13 (90.79)	−2.33(0.27)	707.01 (90.17)

In the "T/Z score" column, data for participants aged ≥ 50 years represent their T-scores, while data for participants aged < 50 years represent their Z-scores. This column displays the combined mean of both scores and is intended primarily to illustrate differences in the relative BMD levels between groups

**Fig. 2:**
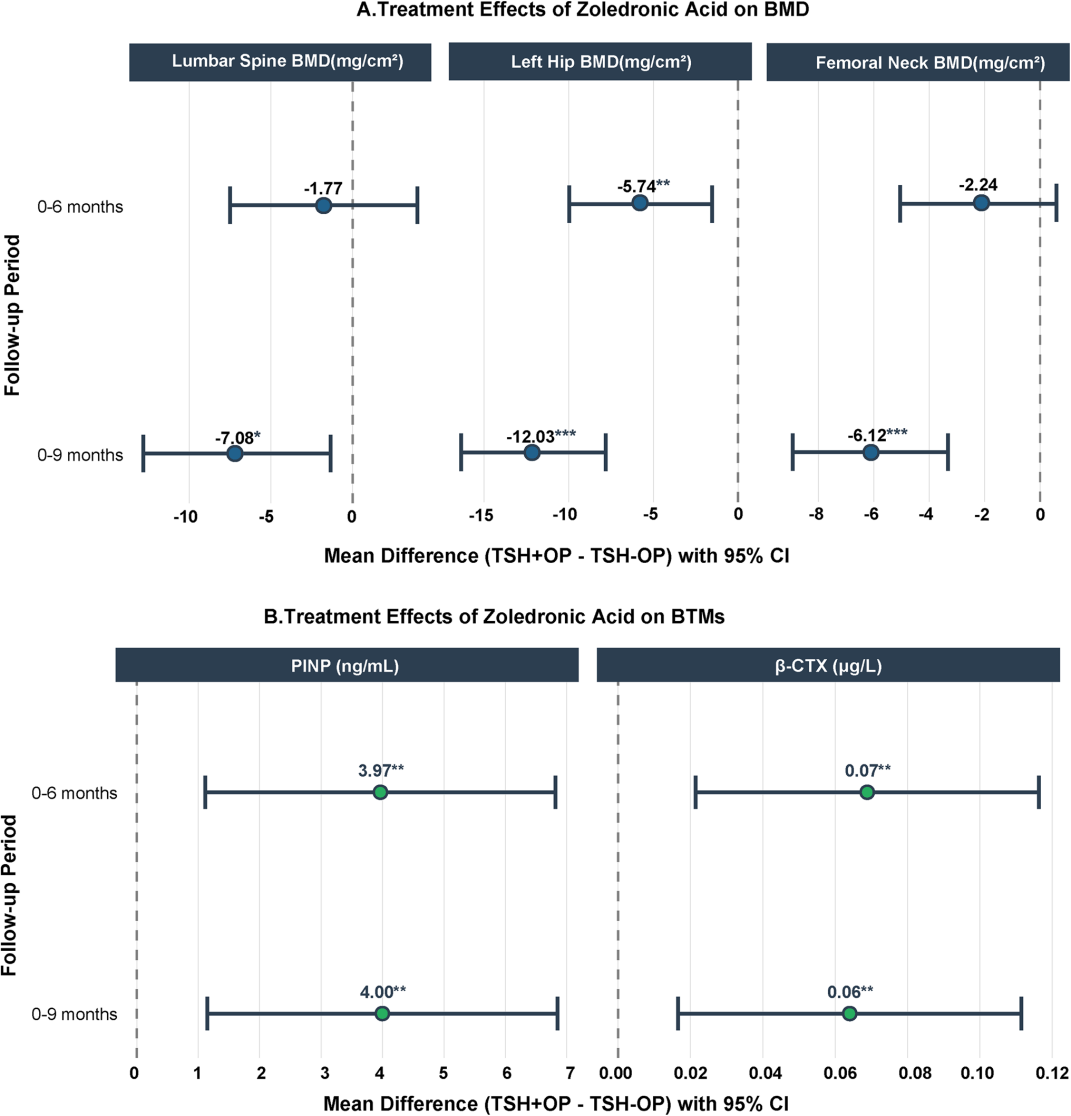
Forest plots display the estimated mean differences in the changes of (**A**) lumbar spine, left hip BMD and femoral neck BMD, and (**B**) BTMs (PINP and β-CTX) between the TSH + OP and TSH − OP groups at 6-month and 9-month follow-ups, derived from linear mixed-effects models adjusted for age, gender, and BMI. Error bars represent 95% confidence intervals. *P < 0.05, **P < 0.01, ***P < 0.001

In the primary analysis, linear mixed-effects model analysis indicated that, after adjusting for age, sex, and BMI, significant differences in BMD changes existed between the TSH + OP and TSH − OP groups. Specifically, at the 6-month follow-up, the intergroup difference in lumbar BMD changes for the TSH + OP group compared to the TSH − OP group was −1.77 mg/cm^2^ (95% CI: −7.50 to 3.96; *P* = 0.54). In contrast, the difference in left hip BMD changes between the groups was −5.74 mg/cm^2^ (95% CI: −9.96 to −1.52; *P* < 0.01) and in femoral neck BMD changes between the groups was −2.24 mg/cm^2^ (95% CI: −5.04 to 0.56; *P* = 0.12). At the 9-month follow-up, the disparities between the groups increased: the difference in lumbar BMD was −7.08 mg/cm^2^ (95% CI: −12.81 to −1.35; *P* = 0.02), in left hip BMD was −12.03 mg/cm^2^ (95% CI: −16.25 to −7.81; *P* < 0.001) , and in femoral neck BMD was −6.12 mg/cm^2^ (95% CI: −8.92 to −3.32; *P* < 0.001) (Fig. 2A).

Regarding bone metabolism markers, the TSH + OP group exhibited elevated bone turnover. Compared with the TSH − OP group, the bone formation marker PINP was 3.97 ng/mL higher at 6 months (95% CI: 1.12 to 6.82; *P* = 0.007) and 4.00 ng/mL higher at 9 months (95% CI: 1.15 to 6.85; *P* = 0.006). Additionally, the bone resorption marker β-CTX was 0.07 µg/L higher at 6 months (95% CI: 0.02 to 0.12; *P* = 0.005) and 0.06 µg/L higher at 9 months (95% CI: 0.02 to 0.11; *P* = 0.008) (Fig. 2B).

**Fig. 3:**
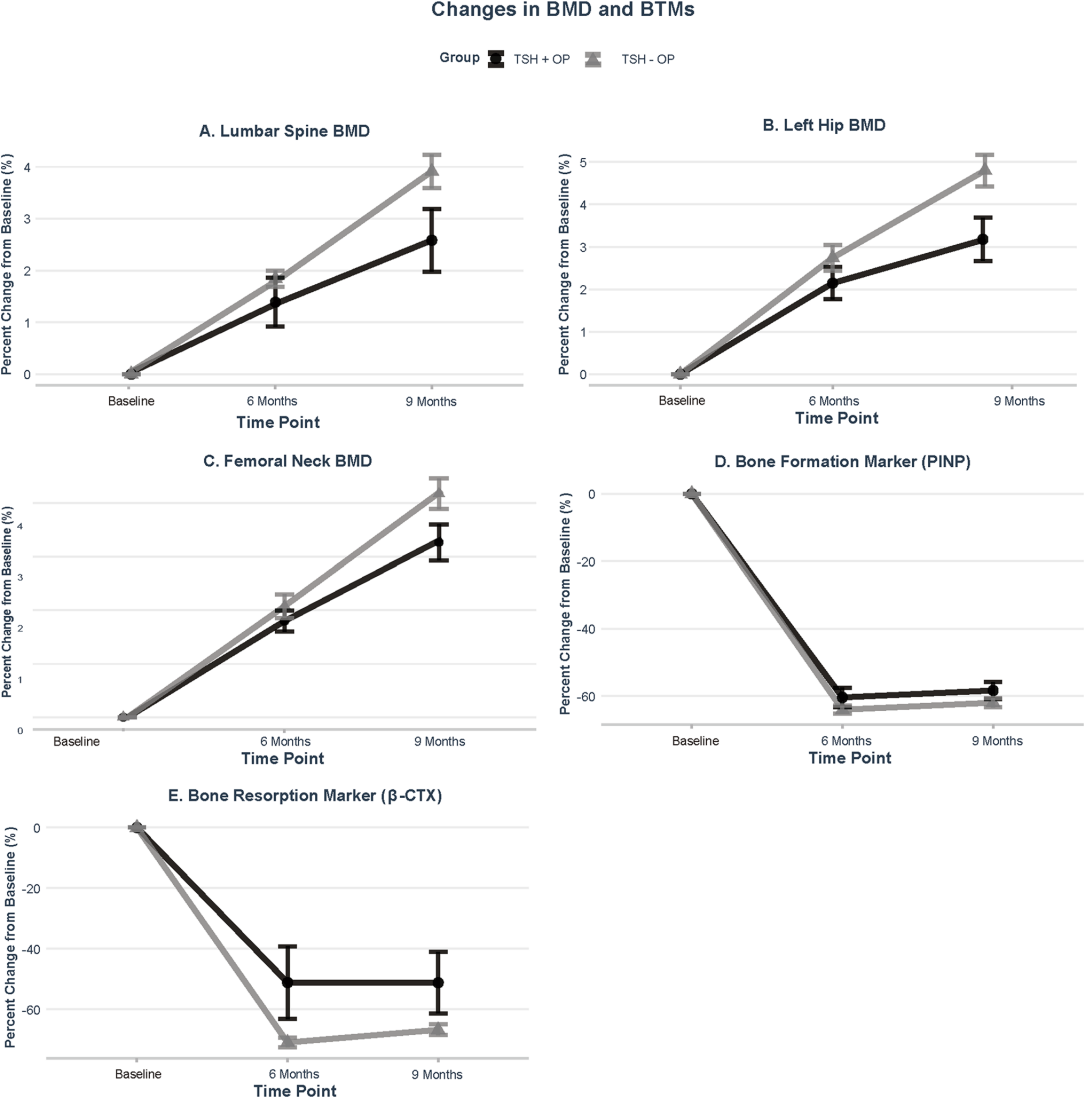
Line graphs depict the percentage changes from baseline at 0 (baseline), 6, and 9 months time points for (**A**) lumbar spine BMD, (**B**) left hip BMD, (**C**) femoral neck BMD, (**D**) PINP, and (**E**) β-CTX. Data are presented as mean ± SEM. TSH + OP group (black); TSH − OP group (gray)

Mean percentage changes in BMD and BTMs during follow-up are illustrated in Fig. 3. In both groups, zoledronic acid treatment was associated with progressive increases in BMD at the lumbar spine, left hip, and femoral neck, with numerically greater gains observed in the TSH − OP group. Reductions in BTMs were also observed in both groups; however, PINP and β-CTX showed smaller percentage decreases in the TSH + OP group at both 6 and 9 months, suggesting a relatively attenuated response.

**Fig. 4:**
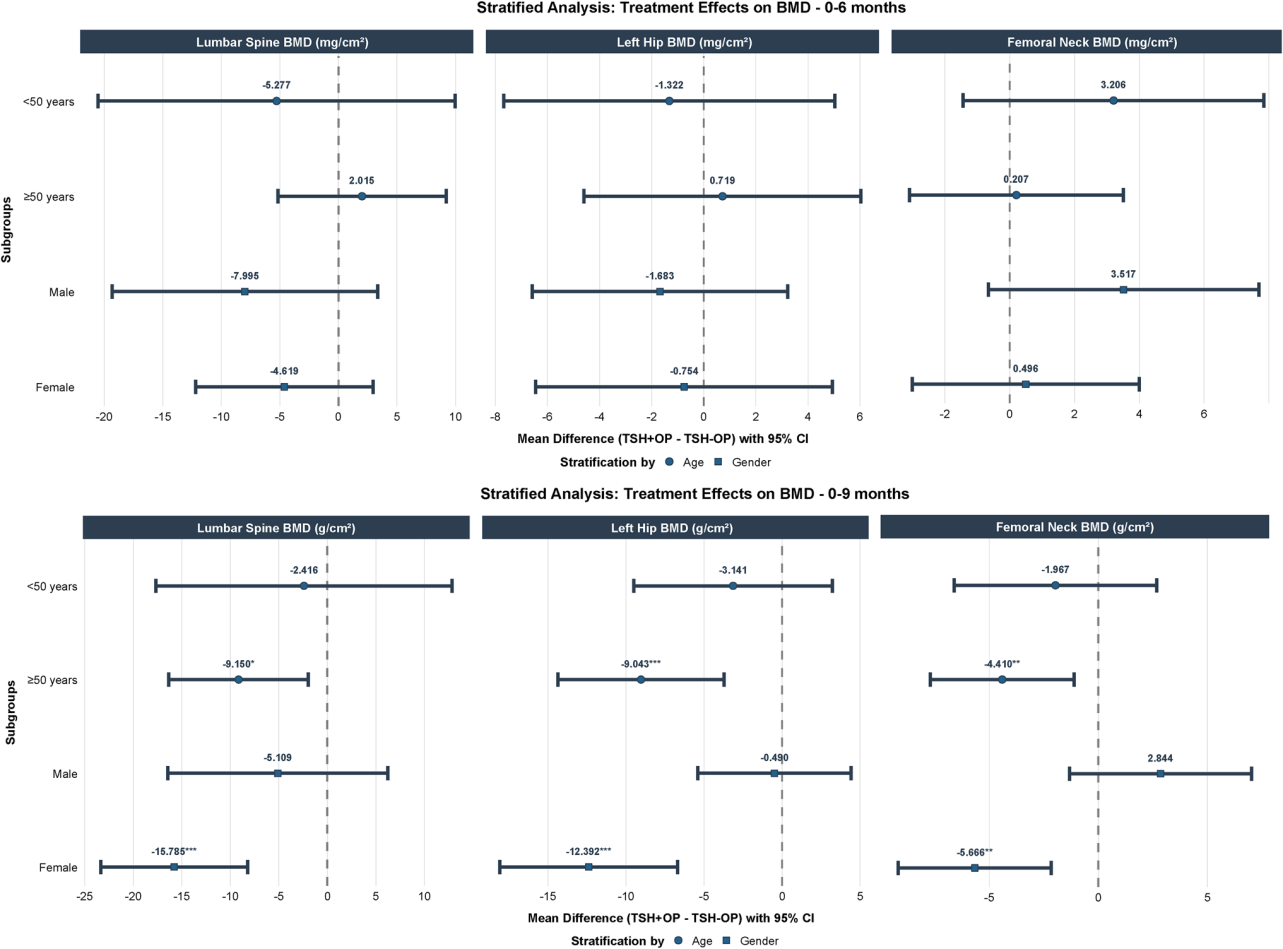
Forest plots display the mean differences in BMD and BTMs changes between groups stratified by age and sex at 6-month and 9-month follow-ups. Analyses were performed using linear mixed-effects models adjusted for age, sex, BMI, smoking, and drinking status. Squares and circles represent point estimates, and horizontal lines indicate 95% confidence intervals. *P < 0.05, **P < 0.01, ***P < 0.001

In exploratory subgroup analyses, differences in BMD changes and BTMs between groups were more evident among participants aged ≥ 50 years and among women, whereas no statistically significant differences were detected in male participants (Fig. 4).

**Fig. 5:**
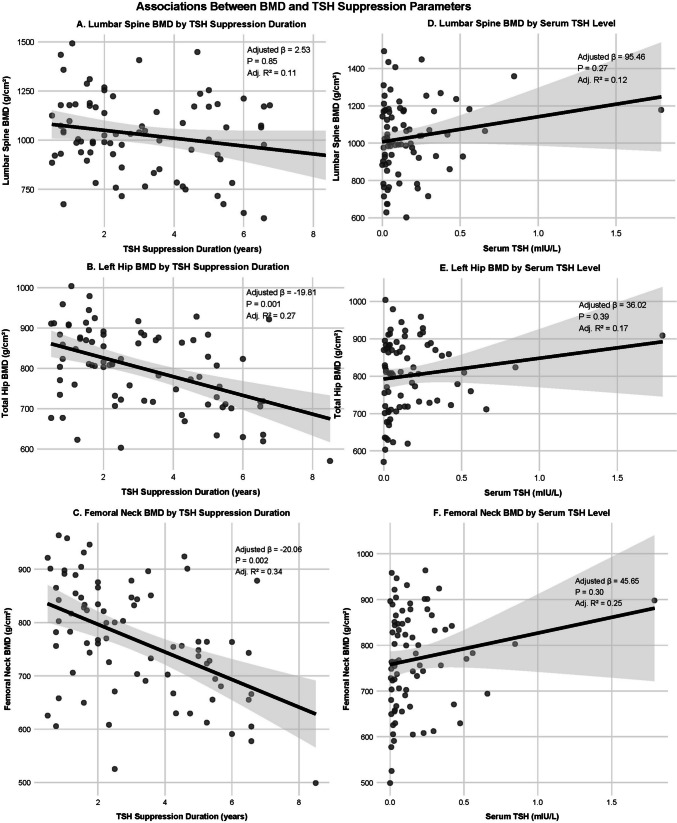
Scatter plots with fitted linear regression lines show the associations of BMD with TSH suppression parameters after adjustment for covariates including age, sex, BMI, smoking, and drinking status. (**A**) Lumbar spine BMD versus TSH suppression duration shows no significant association. (**B**) Left hip BMD versus TSH suppression duration shows a significant negative correlation (adjusted β = −19.81, *P* = 0.001). (**C**) Femoral neck BMD versus TSH suppression duration shows a significant negative correlation (adjusted β = −20.06, *P* = 0.002). (**D**) Lumbar spine BMD versus serum TSH level, (**E**) left hip BMD versus serum TSH level and (**F**) femoral neck BMD versus serum TSH level all show no significant associations. Each panel displays the corresponding adjusted regression coefficient, *P*-value, and adjusted R^2^ value. The gray shading indicates the 95% confidence interval for the regression line.

In exploratory analyses, Pearson correlation analysis demonstrated that TSH suppression duration was negatively correlated with total hip BMD (r = − 0.467, *P* < 0.001) and femoral neck BMD (r = − 0.477, *P* < 0.001), whereas no significant correlation was observed with lumbar spine BMD (r = − 0.201, *P* = 0.075). After adjustment for age, sex, BMI, smoking, and alcohol consumption, partial correlation analyses confirmed a significant negative association between TSH suppression duration and total hip BMD (partial r = − 0.366, *P* = 0.001) as well as femoral neck BMD (partial r = − 0.357, *P* = 0.002), while no significant association was observed for lumbar spine BMD (partial r = 0.023, *P* = 0.848). Consistently, multiple linear regression analysis revealed a significant negative correlation between TSH suppression duration and left hip BMD after adjusting for covariates (β = −19.81, R^2^ = 0.27, *P* = 0.001), indicating a 19.81 mg/cm^2^ BMD reduction per additional suppression year. Similarly, TSH suppression duration demonstrated a significant negative correlation with femoral neck BMD (β = −20.06, R^2^ = 0.34, *P* = 0.002), indicating a 20.06 mg/cm^2^ BMD reduction per additional suppression year. No significant associations were found between TSH suppression duration and lumbar BMD, or between serum TSH levels and BMD at either site (Fig. 5).

## Discussion

In this prospective cohort study, we demonstrate that TSH suppression therapy is associated with a significantly attenuated skeletal response to early zoledronic acid, as evidenced by smaller gains in BMD and persistently elevated BTMs (PINP and β-CTX). These findings extend previous observations by suggesting that TSH suppression may modify the early pharmacological response to anti-resorptive therapy, an aspect that has received limited attention in prior clinical studies, which have largely focused on baseline bone loss rather than treatment response [8, 9, 16].

A substantial body of observational evidence and meta-analyses has demonstrated that long-term TSH suppression is associated with reduced BMD, particularly in postmenopausal women, whereas skeletal effects in men and premenopausal women are less consistent [17, 18]. The systematic review and meta-analysis by Ku et al. reported significant reductions in lumbar spine and total hip BMD among postmenopausal women receiving suppressive levothyroxine therapy [9]. Similarly, Lee et al. observed lower femoral neck BMD and an increased risk of osteoporotic fractures in patients undergoing chronic TSH suppression [19]. These studies established TSH suppression as a clinically relevant risk factor for skeletal fragility but did not address whether pharmacological osteoporosis treatment could adequately counteract this risk. Our findings add to this literature by demonstrating that early BMD gains following zoledronic acid treatment are attenuated in patients with suppressed TSH. This observation is consistent with earlier reports suggesting reduced responsiveness to bisphosphonate therapy in the context of TSH suppression [10], and it may help explain why fracture risk remains elevated in certain populations of differentiated thyroid cancer despite adherence to guideline-recommended osteoporosis management [19–22].

The attenuated therapeutic response observed in this study is biologically plausible when considering the effects of thyroid-related hormonal alterations on bone remodeling. Excess thyroid hormone accelerates bone turnover by increasing osteoclast activity and shortening the remodeling cycle, resulting in net bone loss [23, 24]. In addition, experimental studies have demonstrated that TSH itself plays a regulatory role in skeletal remodeling. Expression of the TSH receptor on osteoblasts and osteoclast precursors suggests that TSH signaling can inhibit osteoclastogenesis and support osteoblast function [25]. Animal models further indicate that loss of TSH signaling exacerbates bone loss under hyperthyroid conditions, highlighting the contribution of suppressed TSH to increased skeletal remodeling [26]. Experimental evidence demonstrates that TSH directly promotes the proliferation and differentiation of osteoblasts by upregulating the expression of key osteogenic genes such as ALP, BMP2, COL1, and Runx2, while thyroid hormones (e.g., T3) synergize with osteogenic signaling pathways like BMP9/AMPK/p38 to potentiate mesenchymal stem cell osteogenesis and bone formation [27, 28]. Although these mechanistic findings are primarily derived from preclinical models, they provide a plausible support for interpreting the persistently elevated BTMs observed in our TSH-suppressed patients despite bisphosphonate therapy.

An important clinical observation in this study is the site-specific attenuation of BMD response, with more pronounced effects at the hip and femoral neck compared with the lumbar spine. Previous clinical and imaging studies suggest that skeletal involvement under TSH suppression may be site-specific rather than uniform, with cortical-rich regions being particularly vulnerable [29, 30]. Assessments incorporating trabecular bone score and femoral geometry further show that lumbar spine BMD may remain relatively preserved despite deterioration in other skeletal compartments, potentially leading to underestimation of fracture risk when spine BMD is considered in isolation [16, 26]. Given the substantial morbidity and mortality associated with hip fractures, these site-specific findings have important clinical implications. They suggest that standard anti-resorptive therapy may be insufficient to fully mitigate fracture risk at clinically relevant skeletal sites in patients receiving long-term TSH suppression, underscoring the importance of multi-site BMD assessment.

Our subgroup analyses revealed that the attenuating effect of TSH suppression on zoledronic acid efficacy was most pronounced in women aged ≥ 50 years, with no statistically significant effect detected in male participants. This sex- and age-specific pattern aligns with the established understanding of osteoporosis pathophysiology and the skeletal effects of thyroid hormones. The postmenopausal state, characterized by estrogen deficiency, is a period of accelerated bone loss due to increased osteoclast activity and bone turnover [31]. The superimposition of a hypermetabolic state induced by TSH suppression likely creates a synergistic deleterious effect on the skeleton, thereby overwhelming the anti-resorptive capacity of a standard bisphosphonate dose. In contrast, the preserved gonadal hormone levels in premenopausal women and men may provide a protective buffer against thyroid hormone-driven bone loss, potentially explaining the attenuated or absent differential response in these groups. Given the exploratory nature of these subgroup analyses, these findings should be interpreted cautiously.

From a clinical management perspective, our results highlight several considerations for patients with DTC. First, the duration and intensity of TSH suppression should be viewed not only as determinants of baseline skeletal risk but also as potential modifiers of treatment response [20, 21]. Second, BTMs may serve as useful adjuncts for monitoring therapeutic efficacy. Previous studies in general osteoporosis populations have shown that persistently elevated BTMs after anti-resorptive therapy are associated with suboptimal BMD responses [32, 33], and our findings suggest that similar monitoring strategies may be informative in patients undergoing TSH suppression therapy.

Finally, these observations raise the possibility that alternative or adjunctive osteoporosis treatment strategies, as well as refined fracture risk assessment approaches incorporating thyroid-related parameters, may be necessary especially for postmenopausal women. Recent efforts to adapt fracture risk prediction models for patients receiving TSH suppression highlight the need for further prospective validation in this specific clinical context [22].

## Strengths and limitations

The primary strength of this study lies in its prospective design, early and intensive assessment timepoints, and the simultaneous evaluation of dynamic changes in both BMD and BTMs. This approach enabled the capture of early treatment-related dynamics in BMD and BTMs. However, several limitations should be acknowledged. First, the 9-month follow-up period is insufficient for evaluating long-term clinical endpoints such as fracture incidence. Although 12-month outcomes are standard for zoledronic acid, the present study was designed to capture early skeletal dynamics, and longer-term efficacy warrants confirmation in future studies. Additionally, certain subgroup analyses, particularly those involving young men, were restricted by small sample sizes, which may have reduced statistical power. Future studies with larger cohorts and longer follow-up durations are warranted to validate these findings.

## Conclusion

In patients undergoing TSH suppression therapy following surgery for differentiated thyroid cancer, TSH suppression was associated with an attenuated early skeletal response to zoledronic acid compared with patients with osteoporosis without TSH suppression, with this attenuation being more pronounced at the hip and femoral neck.

Serum PINP and β-CTX serve as effective biomarkers for early monitoring of treatment efficacy. Furthermore, with prolonged TSH suppression, regular BMD monitoring, particularly at clinically relevant fracture sites, becomes increasingly important.

## Data Availability

The datasets generated and/or analyzed during the current study are not publicly available due to patient privacy but are available from the corresponding author on reasonable request.

## Declarations

### Clinical trial number

ChiCTR2500112609.
